# Effects of different media supplements on the production of an active recombinant plant peroxidase in a *Pichia pastoris* Δ*och1* strain

**DOI:** 10.1080/21655979.2015.1036208

**Published:** 2015-04-02

**Authors:** Christoph Gmeiner, Oliver Spadiut

**Affiliations:** Vienna University of Technology; Institute of Chemical Engineering; Research Area; Biochemical Engineering; Vienna, Austria

**Keywords:** heme precursor, media supplements, OCH1, *Pichia pastoris*, plant peroxidase, protease inhibitor

## Abstract

Recombinant protein production in microorganisms is one of the most studied areas of research in biotechnology today. In this respect the yeast *Pichia pastoris* is an important microbial production host due to its capability of secreting the target protein and performing posttranslational modifications. In a recent study, we described the development of a robust bioprocess for a glyco-engineered recombinant *P. pastoris* strain where the native α-1,6-mannosyltransfrease OCH1 was knocked out (Δ*och1* strain). This strain produced the glycosylated enzyme horseradish peroxidase (HRP) with more homogeneous and shorter surface glycans than the respective benchmark strain. However, the recombinant Δ*och1* strain was physiologically impaired and thus hard to cultivate. We faced cell cluster formation, cell lysis and consequent intensive foam formation. Thus, we investigated the effects of the 3 process parameters temperature, pH and dissolved oxygen concentration on (1) cell physiology, (2) cell morphology, (3) cell lysis, (4) productivity and (5) product purity in a multivariate manner. However, not only process parameters might influence these characteristics, but also media supplements might have an impact. Here, we describe the effects of different heme-precursors as well as of a protease-inhibitor cocktail on the production of active HRP in therecombinant *P. pastoris* Δ*och1*strain.

## Abbreviations

HRP: horseradish peroxidase
OCH1, α-1: 6-mannosyltransfrease Outer CHain elongation 1
Δ*och1*: *P. pastoris* strain with OCH1 knockout

## Introduction

*Pichia pastoris*, a methylotrophic yeast, is widely used for recombinant protein production due to the possibility of reaching high cell densities during cultivation, protein segregation and the capability of performing posttranslational modifications (e.g.^1-3^). However, a significant disadvantage of this microorganism is its tendency to perform hyperglycosylation of proteins.^4^ This represents a significant problem for the production of biopharmaceuticals and for subsequent downstream processing.^5,6^

Strain engineering of *P. pastoris* depicts an interesting option to conquer the problem of hyperglycosylation. In a recent study, we described the characterization of a recombinant *P. pastoris* strain where the native α-1,6-mannosyltransfrease OCH1 was knocked out (Δ*och1* strain;^4^). This Δ*och1* strain, which recombinantly produced the heme-enzyme horseradish peroxidase (HRP;^6^), showed a growth impaired phenotype and considerable rearrangements of cell wall components leading to substrate dependent cell cluster formation and cell lysis. Consequent intensive foam formation made this strain hard to cultivate. In a subsequent study we developed a robust fed-batch process for this recombinant Δ*och1* and investigated the effects of the 3 process parameters temperature, pH and dissolved oxygen concentration on (1) cell physiology, (2) cell morphology, (3) cell lysis, (4) productivity and (5) product purity in a multivariate manner.^7^ We found out that the strain could not be cultivated at 30°C without methanol accumulation and that highest productivity and product purity were reached at 20°C, a pH of 5.0 and a dissolved oxygen concentration of 10%.^7^ However, not only process parameters can influence productivity and product purity, but also media supplements may affect these parameters. Thus we tested potential effects of (1) different heme precursors and (2) a protease inhibitor cocktail on productivity and product purity by adding these compounds to shake flask cultivations of the recombinant Δ*och1* strain.

### Effect of heme precursors

The enzyme HRP comprises 4 disulfid-briges, 2 Ca^2+^- ions as prosthetic group and an iron-protoporphyrin-ring (a heme group) as cofactor in the active site.^8,9^ It is known that the addition of a heme-precursor supports the production of active heme enzymes.^10^ In a recent study, we investigated the effect of adding either the heme-precursors Δ-aminolevulinic acid (ALA and ferric sulfate (FeSO_4_) or hemin on the production of active HRP using a recombinant *P. pastoris* benchmark strain with active OCH1.^11^ We found out that medium supplementation with the traditionally used and pricy heme precursor ALA did not increase the yield of active product. FeSO_4_ and hemin on the other hand turned out to be useful medium supplements to increase the yield of active heme protein.^11^ Thus, we tested these precursors also for the production of HRP with the recombinant Δ*och1* strain.^7^

**Figure 1.**
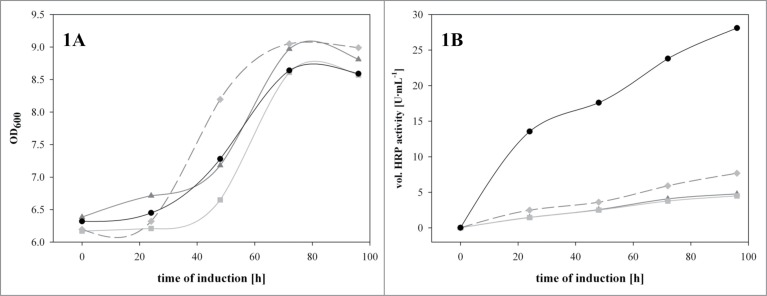
The recombinant Δ*och1* strain was cultivated under inducing conditions in the presence of different heme precursors in shake flasks at 25°C for 96 hours. (**A**) OD_600_ values over induction time; (**B**) volumetric enzyme activity in U·mL^−1^ over induction time. Dark gray solid line with triangles, no heme precursor; light gray solid line with squares, ALA; dark dashed line with diamonds, FeSO_4_; back solid line with dots, hemin.

After overnight cultivation in 5 mL BMGY/Zeocin (1% yeast extract; 2% peptone; 100 mM potassium phosphate buffer, pH 6.0; 1,34% YNB; 4·10^−5^% biotin; 1% glycerol; 50 μg·mL^−1^ Zeocine^TM^) in 100 mL baffled shake flasks at 30°C and 230 rpm, the cell suspension was transferred into 20 mL BMMY/Zeocin (1% yeast extract; 2% peptone; 100 mM potassium phosphate buffer, pH 6.0; 1,34% YNB; 4·10^−5^% biotin; 0.5% methanol; 50 μg·mL^−1^ Zeocine^TM^) in 250 mL baffled shake flasks and cultivated at 20°C and 230 rpm. We reduced the cultivation temperature from 30°C to 20°C upon induction since we had seen highest productivity and purity for the recombinant Δ*och1* strain at the lower temperature previously.^7^ The BMMY/Zeocin contained either no heme-precursor or ALA [1 mM], FeSO_4_ [1 mM] or hemin [30 μM], respectively. Cells were cultivated for 96 hours under these inducing conditions. Every day, samples were taken and analyzed for OD_600_, extracellular protein content and enzymatic HRP activity and 0.5% (v/v) pure methanol was pulsed. In **Figure 1** the time courses of biomass growth (followed by OD_600_ values; **Fig. 1A**) and the extracellular HRP activity (**Fig. 1B**) are shown.

As shown in **Fig. 1A**, the growth of the recombinant Δ*och1* strain was not affected by the presence of either heme precursor. For all the cultivations the strain grew in the first 70 hours of induction, before limitations caused reduced growth. However, in terms of the amount of active extracellular HRP, we observed drastic differences (**Fig. 1B**). The traditionally used and pricy heme precursor ALA did not have any impact on the amount of active HRP but we observed a slight positive effect of FeSO_4­_ (1.6-times more active enzyme). However, we obtained 7-times more active HRP when we induced the recombinant Δ*och1* strain in the presence of 30 μM hemin. In a previous study we observed a boost in the amount of active HRP of up to fold18- when we added 10 μM hemin to minimal media in microscale cultivations of a recombinant *P. pastoris* benchmark strain.^11^ In comparison, in the present study performed with the recombinant Δ*och1* strain we obtained only a 7-fold boost. This discrepancy might be explained by the different media used in the 2 studies. In our previous study we used minimal media, whereas here we used complex BM(G/M)Y medium, which already contained a certain amount of heme-precursors. Thus the observed boost was not that pronounced. However, we can conclude that the boosting effect by hemin is product related and not strain dependent.

Interestingly, FeSO_4_ and hemin did not only affect the enzymatic activity, but also the total amount of extracellular protein. In **Table 1** the values for OD_600_, HRP activity, extracellular protein content and specific activity at the end of cultivation are shown. Clearly we obtained the highest amount of active HRP in the presence of hemin, however we also reached the highest extracellular protein concentration under these conditions. In terms of specific activity, we observed a more than 3-fold higher value for HRP produced in the presence of hemin compared to the traditionally used ALA.

**Table 1:** Values for OD_600_, HRP activity, total protein content and specific activity for the recombinant Δ*och1* strain induced in the presence of different heme precursors for 96 hours

Heme precursor	OD_600_	HRP activity [U· mL^−1^]	Protein content [mg· mL^−1^]	Specific activity [U· mg^−1^]
No precursor	8.81	4.79	0.056	84.8
ALA [1 mM]	8.57	4.51	0.053	85.5
FeSO_4_ [1 mM]	8.99	7.70	0.065	117.9
hemin [30 μM]	8.59	28.1	0.101	278.1

Furthermore, we investigated if the different heme precursors also influenced cell morphology.^12^ We analyzed all the samples under the microscope and with a Malvern Mastersizer but could not determine any effect on cell morphology (data not shown). Thus, we conclude that addition of hemin is an effective strategy to obtain more active recombinant peroxidase not only for the benchmark strain, but also for the growth impaired Δ*och1* strain.

### Effect of protease inhibitor cocktail

**Figure 2.**
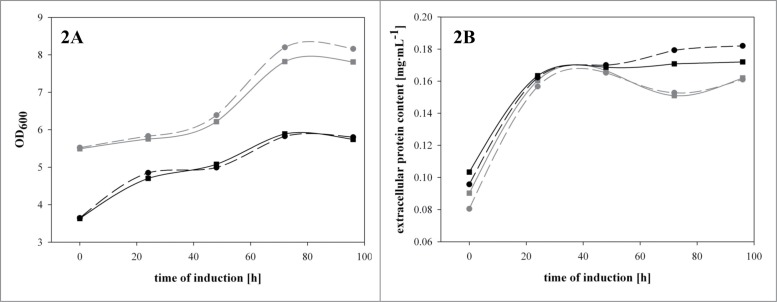
A recombinant benchmark strain and the recombinant Δ*och1* strain were cultivated under inducing conditions in the presence or absence of a protease inhibitor cocktail in shake flasks at 25°C for 96 hours. (**A**) OD_600_ values over induction time; (**B**) total extracellular protein concentration in mg·mL^−1^ over induction time. Dark gray solid line with squares, benchmark strain without protease inhibitor; dark gray dashed line with dots, benchmark strain with protease inhibitor; black solid line with squares, Δ*och1* strain without protease inhibitor; black dashed line with squares, Δ*och1* strain with protease inhibitor.

In our previous studies we had shown that the recombinant Δ*och1* strain was physiologically impaired and strongly affected by cell lysis.^4,7^ It is well known that cell lysis also means the release of intracellular proteases, which could potentially degrade the target product.^13^ Thus, we tested the effect of the presence of a protease inhibitor cocktail (cOmplete Mini EDTA-free^TM^; Roche, Switzerland) on the total amount of extracellular protein and active HRP in shake flask experiments. We conducted this study with the *P. pastoris* benchmark strain with intact OCH1 and with the recombinant Δ*och1* strain. The cultivations were again performed in shake flasks, as described above. Both strains showed growth in the first 70 hours of induction, before limitations caused reduced growth at the later phases of cultivation. However, when we analyzed the growth of these 2 strains during the induction phase in more detail, we clearly confirmed the impaired growth of the recombinant Δ*och1* strain (**Fig. 2A**). Interestingly, when we analyzed the total extracellular protein content (**Fig. 2B**) and the enzymatic HRP activity in the cultivation broths of the 2 strains, cultivated in the presence or absence of the protease inhibitor cocktail, we could not determine any differences. Thus, we concluded that the presence of protease inhibitors does not affect the amount of product for the recombinant benchmark strain nor for the recombinant Δ*och1* strain, respectively. As shown in our previous study, cell cluster formation and consequent cell lysis for the recombinant Δ*och1* strain are C-source dependent and mainly happen in cultivation phases on glycerol – once the cells switch to methanol cell cluster formation as well as lysis are diminished.^7^ As we can see from the present data, it is not necessary to add protease inhibitors to the cultivation broth of the recombinant Δ*och1* strain to protect the product from the proteases released before.

In conclusion we showed that heme precursors can also be used successfully for the recombinant Δ*och1* strain to produce more active HRP. In agreement with our previous study performed with a recombinant benchmark strain,^11^ we found out that addition of hemin gave the highest amount of active product. Furthermore, we showed that endogeneous proteases released by cell lysis events during glycerol cultivation phases of the recombinant Δ*och1* strain do not degrade the product during subsequent induction phases, thus making the addition of protease inhibitors unnecessary.
